# New functions of C3G in platelet biology: Contribution to ischemia-induced angiogenesis, tumor metastasis and TPO clearance

**DOI:** 10.3389/fcell.2022.1026287

**Published:** 2022-10-31

**Authors:** Luis Hernández-Cano, Cristina Fernández-Infante, Óscar Herranz, Pablo Berrocal, Francisco S. Lozano, Manuel A. Sánchez-Martín, Almudena Porras, Carmen Guerrero

**Affiliations:** ^1^ Instituto de Biología Molecular y Celular del Cáncer (IMBCC), USAL-CSIC, Salamanca, Spain; ^2^ Instituto de Investigación Biomédica de Salamanca (IBSAL), Salamanca, Spain; ^3^ Departamento de Angiología y Cirugía Vascular, Hospital Universitario de Salamanca, Universidad de Salamanca, Salamanca, Spain; ^4^ Servicio de Transgénesis, Nucleus, Universidad of Salamanca, Salamanca, Spain; ^5^ Departamento de Medicina, Universidad de Salamanca, Salamanca, Spain; ^6^ Departamento de Bioquímica y Biología Molecular, Facultad de Farmacia, Universidad Complutense de Madrid, Madrid, Spain; ^7^ Instituto de Investigación Sanitaria del Hospital Clínico San Carlos (IdISSC), Madrid, Spain

**Keywords:** C3G, Rap1, platelets, ischemia-induced angiogenesis, platelet-mediated metastasis, thrombopoietin, c-Mpl, RapGEF1

## Abstract

C3G is a Rap1 guanine nucleotide exchange factor that controls platelet activation, aggregation, and the release of α-granule content. Transgenic expression of C3G in platelets produces a net proangiogenic secretome through the retention of thrombospondin-1. In a physiological context, C3G also promotes megakaryocyte maturation and proplatelet formation, but without affecting mature platelet production. The aim of this work is to investigate whether C3G is involved in pathological megakaryopoiesis, as well as its specific role in platelet mediated angiogenesis and tumor metastasis. Using megakaryocyte-specific C3G knockout and transgenic mouse models, we found that both C3G overexpression and deletion promoted platelet-mediated angiogenesis, induced by tumor cell implantation or hindlimb ischemia, through differential release of proangiogenic and antiangiogenic factors. However, only C3G deletion resulted in a higher recruitment of hemangiocytes from the bone marrow. In addition, C3G null expression enhanced thrombopoietin (TPO)-induced platelet production, associated with reduced TPO plasma levels. Moreover, after 5-fluorouracil-induced platelet depletion and rebound, C3G knockout mice showed a defective return to homeostatic platelet levels, indicating impaired platelet turnover. Mechanistically, C3G promotes c-Mpl ubiquitination by inducing Src-mediated c-Cbl phosphorylation and participates in c-Mpl degradation *via* the proteasome and lysosome systems, affecting TPO internalization. We also unveiled a positive role of platelet C3G in tumor cell-induced platelet aggregation, which facilitated metastatic cell homing and adhesion. Overall, these findings revealed that C3G plays a crucial role in platelet-mediated angiogenesis and metastasis, as well as in platelet level modulation in response to pathogenic stimuli.

## Introduction

C3G (RapGEF1) is a ubiquitously expressed GEF (guanine nucleotide exchange factor) with important functions in megakaryocyte (MK) and platelet biology, where it acts primarily through Rap1b GTPase. C3G regulates platelet activation and aggregation in response to most platelet agonists (Gutiérrez-Herrero et al., 2012; Martín-Granado et al., 2017; Gutiérrez-Herrero et al., 2020). It also contributes to MK differentiation and proplatelet formation (Ortiz-Rivero et al., 2018).

Platelets are essential mediators of ischemia-induced neoangiogenesis. In response to hypoxia, VEGF (vascular endothelial growth factor), released by platelets and endothelial cells, induces platelet secretion of SDF-1 (stromal-derived factor-1, also known as CXCL12). VEGF and SDF-1 facilitate the recruitment of proangiogenic progenitor cells (hemangiocytes) from the bone marrow (BM) (Jin et al., 2006; Massberg et al., 2006). Hemangiocytes release angiogenic factors at the ischemic site that promote the incorporation and assembly of endothelial progenitor cells and the stabilization of new blood vessels (Feng et al., 2011; Amano et al., 2015).

Additionally, platelets induce tumor vascularization, thus contributing to tumor growth and metastasis (Li, 2016; Huong et al., 2019). They also promote adhesion of tumor cells to the endothelium of target tissues, facilitating extravasation and homing (Bambace and Holmes, 2011; Li, 2016). Reciprocally, tumor cells secrete cytokines that stimulate thrombopoiesis and contribute to the development of thrombocytosis (Kaser et al., 2001). This phenomenon, known as TCIPA (tumor cell-induced platelet aggregation), allows tumor cells to escape from the immune system (Bambace and Holmes, 2011).

On the other hand, numerous solid tumors generate abnormal thrombopoietin (TPO) levels and other thrombopoietic factors, leading to reactive thrombocytosis (Gastl et al., 1993; Werynska et al., 2003). TPO is constitutively produced in the liver and its plasma concentration inversely correlates with the number of platelets, which remove it by clearance, thus, modulating their own mass (Kaushansky and Drachman, 2002; de Graaf and Metcalf, 2011). In addition, TPO promotes the recovery of hematopoietic stem cell and platelet levels after 5-FU (5-fluorouracil)- or irradiation-induced myelosuppression (Li and Slayton, 2013). In platelets, TPO binds to its receptor, c-Mpl, and induces its endocytosis, recycling and degradation (Hitchcock et al., 2008). This mechanism is driven by the E3 ubiquitin ligase c-Cbl, responsible for the ubiquitination of c-Mpl and its degradation by the proteasome and lysosome systems (Saur et al., 2010; Marklin et al., 2020). C-Cbl is phosphorylated and activated by SFKs (Src family kinases), such as Lyn (Murphy et al., 2013), Fyn (Hunter et al., 1999) and Src (Yokouchi et al., 2001).

It has been proposed that TPO-c-Mpl engages CrkL-C3G-Rap1 signaling pathway to induce sustained activation of ERKs, which is required for MK differentiation (Garcia et al., 2001; Stork and Dillon, 2005). An interaction between C3G and c-Cbl has been detected in K562 cells (Maia et al., 2013), an erythromegakaryoblastic cell line that, upon stimulation with phorbol 12-myristate 13-acetate (PMA), acquires MK markers, including c-Mpl expression (Jacquel et al., 2006; Ortiz-Rivero et al., 2018).

In this work, we have uncovered a new relevant role for C3G in ischemia-induced angiogenesis and tumor metastasis, as well as its participation in platelet recovery following BM depletion or TPO stimulation, through inducing c-Cbl-dependent c-Mpl ubiquitination and degradation.

## Materials and methods

### Mouse models

The transgenic (tgC3G, expressed under the PF4 promoter) and conditional knockout (Rapgef1^flox/flox^;PF4-Cre, hereinafter C3G-KO) mouse models for C3G used in this work have been described and characterized in previous works. Briefly, tgC3G mice show increased platelet activation and aggregation in response to thrombin, ADP, PMA and collagen, accompanied by increased thrombus formation (Gutiérrez-Herrero et al., 2012), while C3G-KO mice show the opposite phenotype (Gutiérrez-Herrero et al., 2020). TgC3G platelets also present alterations in α-granule secretion, with retention of VEGF, bFGF and specially TSP-1, which favors tumor growth and metastasis (Martín-Granado et al., 2017). In addition, transgenic C3G expression under the PF4 promoter favors MK differentiation and proplatelet formation (Ortiz-Rivero et al., 2018). See additional information in Supplementary Table S1.

WtC3G was the control mice for tgC3G mice, while C3G-wt was the control mice for the C3G-KO model. All mice used were 8-12 weeks old.

### Tumor cell implantation-induced ischemia

We followed the protocol previously published (Martín-Granado et al., 2017). Briefly, mice were injected subcutaneously with 5 × 10^5^ 3 LL murine Lewis lung carcinoma cells, diluted in 100 μl of PBS. Tumors were harvested by detaching the surrounding connective tissue 15 days after implantation, weighed, and processed for immunohistochemistry.

### Hindlimb ischemia

A unilateral hindlimb ischemia was generated following the protocol described in (Feng et al., 2011). Blood flow was monitored by Laser Doppler 0, 1, 2 and 14 days post-surgery, as described in Niiyama et al. (2009).

### Detection of hemangiocytes

EDTA-anticoagulated blood samples or BM cells were incubated with anti-CXCR4-PE and anti-VEGFR-APC antibodies and analyzed by flow cytometry, as described (Feng et al., 2011). Data are presented as percentage of hemangiocytes in the total blood cell count.

### Platelet and MK purification and detection

Platelets were purified from blood collected by cardiac puncture, and counted by flow cytometry as previously described (Gutiérrez-Herrero et al., 2020). Bone marrow cells (BMCs) were obtained from the femora and tibiae of mice and MKs were purified by BSA density gradient as described (Gutiérrez-Herrero et al., 2020). Alternatively, MKs were obtained *in vitro* by culturing BMCs in RPMI with 10% horse serum, supplemented with 50 ng/ml recombinant TPO, 10 ng/ml IL-3 (Interleukin-3, Invitrogen), 10 ng/ml SCF (Stem Cell Factor), 10 ng/ml IL-11 and 10 ng/ml IL-6 (Miltenyi) for 6 days. MKs were detected by flow cytometry with anti-CD41-FITC (MWReg30) and anti-CD61-PE (2C9.G3) antibodies, or anti-CD42b-FITC antibody, previous gate with anti-CD61-PE antibody. Ploidy analysis was performed as described (Ortiz-Rivero et al., 2018; Gutiérrez-Herrero et al., 2020).

### Gene expression analysis in tissue homogenates

For RT-qPCR analysis of VEGFA, CD31 and SDF-1 (muscle) or TPO (liver), total RNA was isolated from 50 mg of tissue in NZYol (NZYTech), using a GentleMac Dissociator (Miltenyi). Single-strand cDNA was generated with NZY First-Strand cDNA Synthesis Kit (NZYTech), according to the manufacturer’s instructions. qPCR was performed in triplicate. Gene expression results were normalized to β-actin (ACTB). Primer sequences are depicted in Supplementary Table S2.

### Histology and immunohistochemistry

Vessel formation was quantified morphologically by immunohistochemical detection with anti-CD31 antibody (Abcam, ab28364) in paraffin embedded sections of tumors or ischemic skeletal muscle, as previously described (Martín-Granado et al., 2017).

### Analysis of platelet releasate

Platelets (1 × 10^9^), were stimulated with 0.2 U/ml thrombin and releasate purified as previously described (Martín-Granado et al., 2017). The level of SDF-1 in the releasate was measured using the Proteome Profiler Mouse Angiogenesis Array Kit (R&D Systems), following the manufacturer´s instructions.

### Capillary tube formation assay

Angiogenic capacity of secretomes was determined using Ibidi µ-Slide Angiogenesis (Ibidi 81506) plates by analyzing three parameters: 1) number of master segments (segments bounded by two branches), 2) total length of master segments and 3) number of internal meshes. These parameters were acquired with the Skeleton Analyzer plug-in (Arganda-Carreras et al., 2010) and the Angiogenesis Analyzer Macro (Carpentier G, Angiogenesis Analyzer for ImageJ (2012) available online: http://imagej.nih.gov/ij/macros/toolsets/Angiogenesis%20Analyzer.txt).

### Short-term metastasis

Mice were injected retro-orbitally with 2 × 10^6^ GFP-expressing B16-F10 melanoma cells in 150 μl PBS, and killed 1 h after injection. The left lung lobes were digested 45 min at 37°C with a mixture of collagenase/dispase (0.1 mg/ml) and cell suspension filtered through a 70 μm strainer. Viable GFP-positive tumor cells were counted per 1,000,000 lung cells by flow cytometry. The right lung lobes were fixed and sections assessed microscopically by immunofluorescence or immunohistochemistry with anti-GFP (FL) antibodies.

### Adhesion of tumor cells

Adhesion of B16-F10 cells, incubated with platelets, to poly-L-lysine-coated glass coverslips was performed as described (Becker et al., 2017). Fixed cells were stained with anti-P-selectin + anti-goat Cy3 antibodies and phalloidin. Cells on the coverslip were analyzed with a Leica SP8 fluorescence microscope.

### Platelet activation and aggregation

Platelet activation was determined by flow cytometry *via* measuring the high-affinity conformation of the integrin αIIbβ3 with Alexa Fluor^TM^-488-labeled fibrinogen, as described (Gutiérrez-Herrero et al., 2012). Platelet aggregation was induced by the addition of 3 × 10^4^ B16-F10 cells (in 30 μl) and determined by flow cytometry as described (Gutiérrez-Herrero et al., 2020).

### Platelet and MK production *in vivo*

Mouse TPO (Innovative Research) was administered intravenously (0.5 μg per mouse in 100 μl PBS). Platelet number was determined at the indicated time points. BMCs were harvested at the final point of the experiment and the percentage of MKs analyzed by flow cytometry with anti-CD41-FITC and anti-CD61-PE antibodies.

### Depletion of cycling hematopoietic cells

Based on (Li and Slayton, 2013), mice were injected intraperitoneally with 150 mg/kg 5-fluorouracil (5-FU, Merck) and platelet rebound monitored at days 0, 4, 7, 10, 14, 18, and 21 post-injection. 5-FU was prepared in 0.9% NaCl containing 6.75% DMSO.

### c-Mpl surface expression and internalization

Analysis of c‐Mpl levels on the surface was performed by flow cytometry in non-stimulated or TPO-stimulated platelets (25 ng/ml, 30 min), as described (Marklin et al., 2020), using anti-c-Mpl antibody (AMM2 clone), followed by staining with anti-CD41-FITC and anti-rat Cy5 antibodies.

### TPO measurements

Plasma was obtained from individual mice using Microvette^®^ 500 Serum Gel (Sarstedt), and TPO levels were measured by ELISA (Quantikine, R&D). *In vitro* detection of TPO uptake was performed after 30 min incubation, as described (Marklin et al., 2020).

### Confocal immunofluorescence microscopy

Platelets from three mice were pooled and activated with agonists (0.5 U/ml thrombin, 1 min; 25 μM ADP, 5 min or 25-100 ng/ml TPO, 5 min). Inhibitors (10 μM PP2, 30 μM MG132 or 20 mM NH_4_Cl) were added prior agonists (5 min PP2, 1 h MG132 and NH_4_Cl). Platelets were fixed and permeabilized as previously described (Gutiérrez-Herrero et al., 2020). Incubation with primary antibodies against VEGF, TSP-1, SDF-1, C3G (Guerrero et al., 1998), c-Cbl, phospho-c-Cbl, phospho-Src Y418, c-Mpl and Ubiquitin was performed at RT for 2 h, followed by incubation (1 h at RT) with secondary antibodies: Alexa Fluor™-568-conjugated goat anti rabbit and Alexa Fluor™-647-conjugated goat anti mouse. Phalloidin was used for actin detection. Antibody details are depicted in Supplementary Table S3.

Immunofluorescence was quantified with ImageJ software. Colocalization was determined by Pearson´s Correlation Coefficient (P) analysis, using ImageJ with the Coloc2 plugin, as described (Peters et al., 2012).

### Western blot and immunoprecipitation

Platelet and MK protein extracts were prepared by lysing cells directly in sample loading buffer, as described (Shankar et al., 2006).

For immunoprecipitation, platelets were lysed in standard RIPA buffer (Martín-Granado et al., 2017). Immunocomplexes were pulled-down with anti-C3G (G-4) antibody and purified with protein G agarose resin four rapid run (ABT). Antibodies used for western blot were against: VEGF, TSP-1, c-Cbl, Rap1, c-Mpl, β-actin and β-tubulin (details in Supplementary Table S3).

### Rap1 activation assay

The activated, GTP-bound form of Rap1 was pulled-down using GST-RalGDS RBD immobilized on glutathione-sepharose beads, as described previously (Gutiérrez-Herrero et al., 2012).

### Statistics

Data are represented as the mean ± SD (standard deviation) or SEM (standard error of the mean) as indicated. The Kolmogorov-Smirnov test was performed to determine if data fit a normal distribution. To compare two experimental groups for which the data were normally distributed, the unpaired Student’s t-test was carried out. The nonparametric Mann–Whitney *U*-test was carried when the data were not normally distributed. Differences were considered significant when *p* < 0.05.

Antibodies used for flow cytometry, confocal immunofluorescence microscopy, immunohistochemistry, immunoprecipitation and western blot are listed in Supplementary Table S3.

## Results

### Platelet C3G regulates ischemia-induced angiogenesis

**FIGURE 1:**
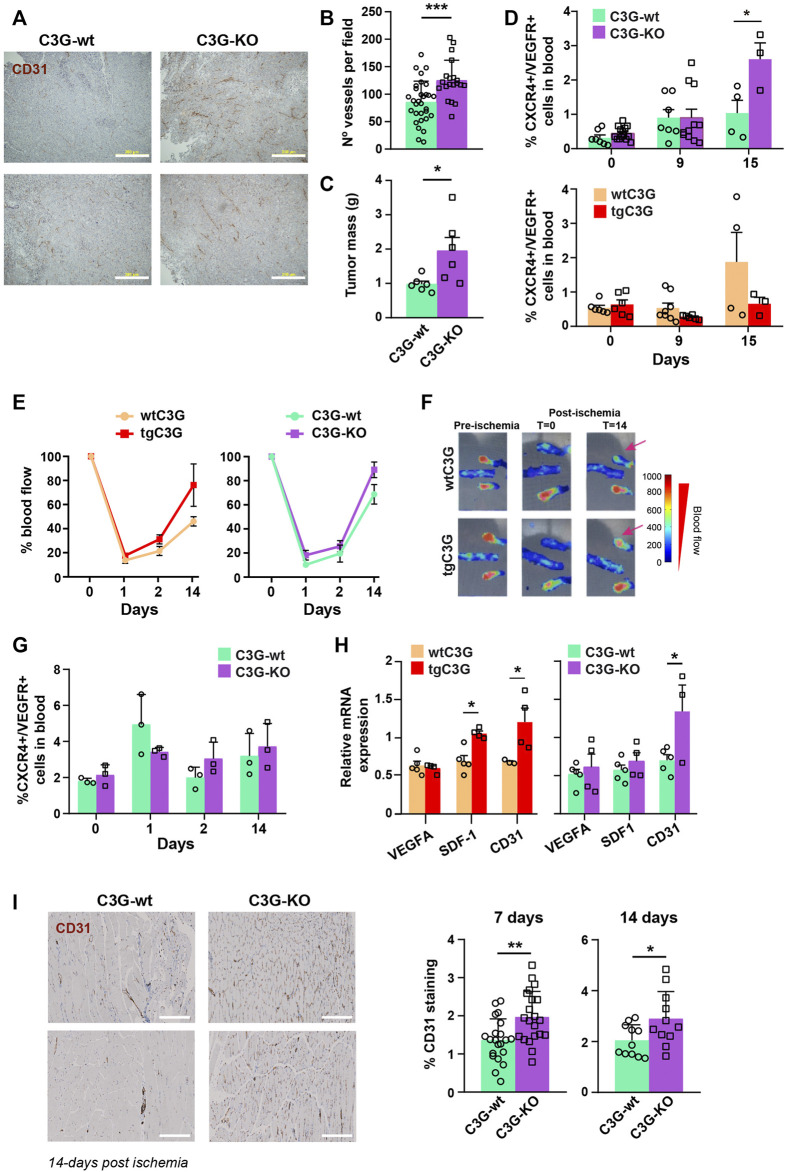
Platelet C3G regulates ischemia-induced angiogenesis. **(A)** Representative images of 3 LL tumor sections, from C3G-wt and C3G-KO mice, showing vessel density detected by CD31 staining. Bar: 200 μm. **(B)** Quantification of the number of vessels per field (0.65 mm^2^) in tumor sections (Mean ± SEM). **(C)** Mean ± SEM of the tumor mass (in grams). **(D)**. Mean ± SEM of the percentage of hemagiocytes (CXCR4/VEGFR double-positive cells). **(E)** Doppler values represented as the mean ± SEM of the percentage of blood flow (n = 6 tgC3G and wtC3G, n = 3 C3G-KO and C3G-wt). **(F)** Representative Doppler images at the indicated times. The color scale represents the blood flow values. **(G)** Mean ± SEM of the percentage of CXCR4/VEGFR-double positive cells in blood from C3G-KO and control mice at the indicated times post-ischemia. **(H)** RT-qPCR analysis of VEGFA, SDF-1 and CD31 mRNA expression in homogenates of murine muscle from the indicated genotypes at day 14 post-ischemia. Values are relative to β-actin expression and were normalized against the corresponding value in the contralateral (non-ischemic) leg. **(I)** Left: representative images of ischemic leg sections of C3G-wt and C3G-KO mice, showing vessel density detected by CD31 staining. Bar: 200 μm. Right: mean ± SD of the percentage of positive pixels for CD31 staining, relative to the total number of pixels. *p < 0.05; **p < 0.01; ***p < 0.001.

Our previous published data showed that transgenic expression of C3G in platelets induces neovascularization in two models of syngeneic heterotopic tumor cell transplantation: murine Lewis lung carcinoma (3LL) and B16-F10 mouse melanoma cells (Martín-Granado et al., 2017). Interestingly, we have now found that ablation of C3G in platelets also resulted in larger and more vascularized 3LL tumors (Figures 1A–C and Supplementary Figure S1A). Fast tumor growth generates hypoxia, which promotes the release of SDF-1 from platelets and the subsequent recruitment of CXCR4^+^/VEGFR^+^ cells (hemangiocytes) from the BM (Jin et al., 2006; Feng et al., 2011). To analyze if this mechanism is behind the role of platelet C3G in angiogenesis, we measured the presence of hemangiocytes in peripheral blood (PB) of tgC3G and C3G-KO mice and their controls upon implantation of 3LL cells. C3G-KO mice showed a significant increase in the percentage of hemangiocytes at day 15 after implantation (Figure 1D; Supplementary Figure S1B), which correlates with *in vivo* tumor growth. In contrast, recruitment of hemangiocytes to PB tends to decrease in tgC3G mice, especially at day 15 (Figure 1D; Supplementary Figure S1B).

To corroborate these findings, we analyzed angiogenesis in a model of hindlimb ischemia. As shown in Figures 1E,F, blood flow was recovered slightly faster in both tgC3G and C3G-KO mice, compared to their controls. However, as in the tumor ischemia model, only in C3G-KO mice this correlated with a slightly higher recruitment of hemangiocytes at days 2 and 14 (Figure 1G; Supplementary Figure S1C).

SDF-1 is also produced by the hypoxic tissues (Feng et al., 2011). Hence, there was a significant increase in SDF-1 levels in the ischemic muscle of tgC3G animals (day 14 post-ischemia) (Figure 1H). This is in agreement with the increased expression of CD31 (Figure 1H) and could explain the faster recovery of blood flow in these animals. A significant increased CD31 expression was also found in the ischemic muscle of C3G-KO mice (Figure 1H), in agreement with its greater vascularization (Figure 1I).

### Transgenic C3G promotes the retention of SDF-1 in platelets, while deletion of C3G enhanced SDF-1 and VEGF release and TSP-1 retention in response to thrombin

**FIGURE 2:**
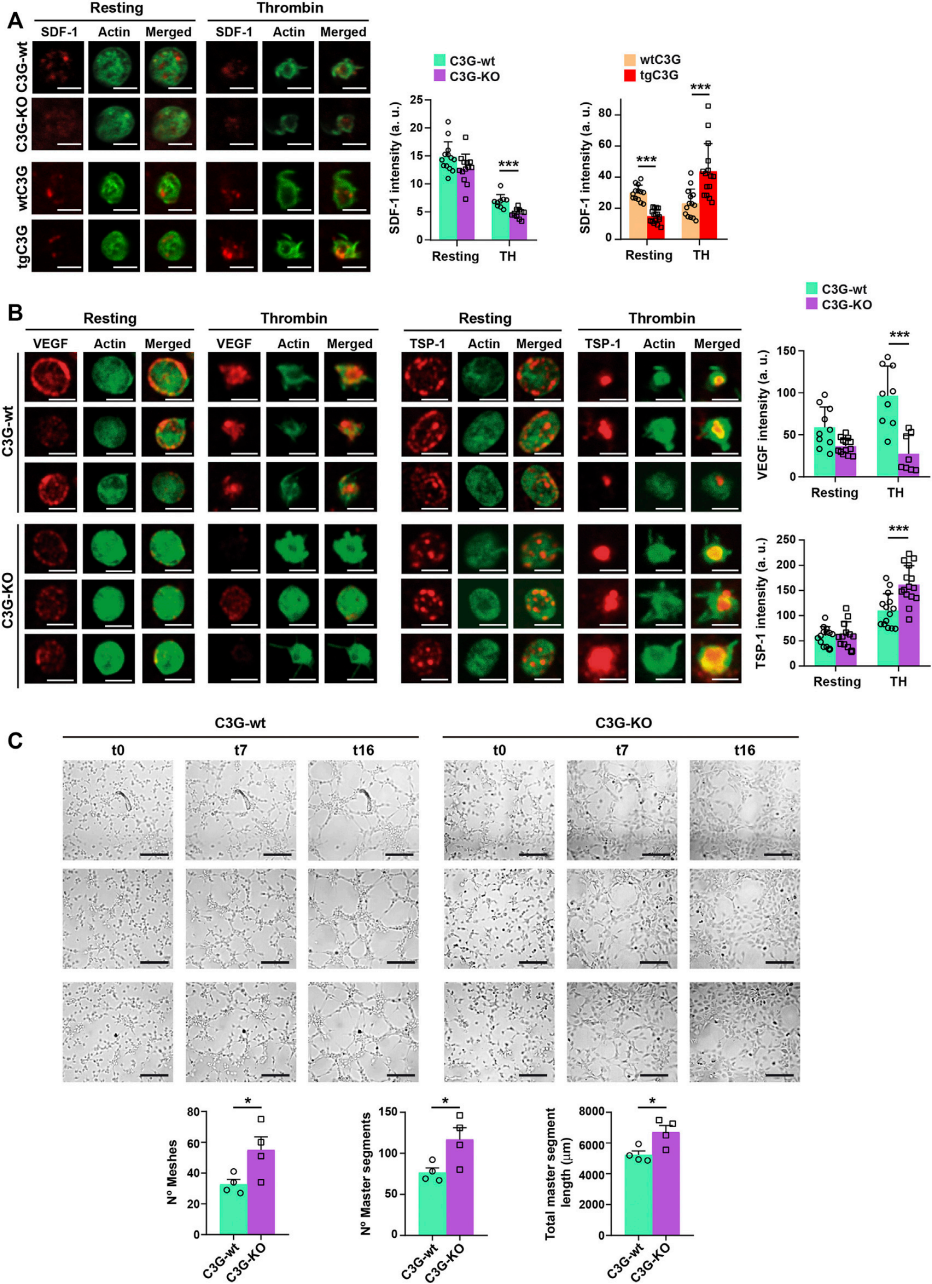
C3G deletion enhances SDF-1 and VEGF release, while C3G overexpression retains SDF-1. **(A)** Representative immunofluorescence images of tgC3G, C3G-KO and control platelets, untreated or treated with thrombin (TH, 0.5 U/ml, 5 min) and stained with anti-SDF-1 + Alexa Fluor™-647 (red) and phalloidin-488 (green), taken at the same exposure time. Bar: 2.5 µm. Histograms represent the mean ± SD of SDF-1 fluorescence intensity. **(B)** Representative immunofluorescence images of C3G-KO and control platelets untreated or treated with thrombin (TH, 0.5 U/ml, 5 min) and stained with anti-TSP-1 + Alexa Fluor™-647 (red) or anti-VEGF + Alexa Fluor™-647 (red) and phalloidin-488 (green). All images were taken at the same exposure time. Bar: 2.5 μm. Histograms represent the mean ± SD of VEGF (upper) and TSP-1 (lower) fluorescence intensity. **(C)** Representative images showing the capillary-like structures formed by HUVECs supplemented with releasates from thrombin-stimulated C3G-KO and C3G-wt platelets. Images were taken every 15 min for 17 intervals. Intervals t0, t7 and t16 are shown, t0 corresponds to 1.5 h after releasate supplementation. Bar: 200 μm. Graphics show the mean ± SEM of different network characteristics determined at 2.5 h. Two independent experiments were performed. *p < 0.05; ***p < 0.001. a.u, arbitrary units.

The above results suggest that C3G could regulate the release of SDF-1 from platelets in response to distant hypoxia. Indeed, SDF-1 content was reduced in thrombin-stimulated C3G-KO platelets, indicating a higher release, while the opposite was found in tgC3G platelets (Figure 2A). In agreement, higher levels of SDF-1 were found in thrombin-induced secretomes from C3G-KO platelets, while slightly lower amount of SDF-1 was found in tgC3G platelet secretomes (Supplementary Figure S1D). These results suggest that C3G plays a negative role in SDF-1 secretion, in parallel with the retention of VEGF and bFGF in thrombin-stimulated tgC3G platelets previously described (Martín-Granado et al., 2017). In addition, deletion of C3G increased VEGF release and TSP-1 retention in response to thrombin (Figure 2B). Accordingly, lower levels of VEGF were found in the cytosolic fraction of C3G-KO platelets, while a higher accumulation of TSP-1 was observed in the plasma membrane (Supplementary Figure S1E). Supporting these findings, thrombin-induced secretome from C3G-KO platelets showed an increased ability to induce capillary-tube formation in HUVEC (Figure 2C), similarly to tgC3G platelets (Martín-Granado et al., 2017).

All these data back up the proangiogenic effect of both tgC3G and C3G-KO platelets seen in the *in vivo* ischemia models.

### Platelet C3G stimulates short-term melanoma metastasis through regulation of melanoma cell adhesion

**FIGURE 3:**
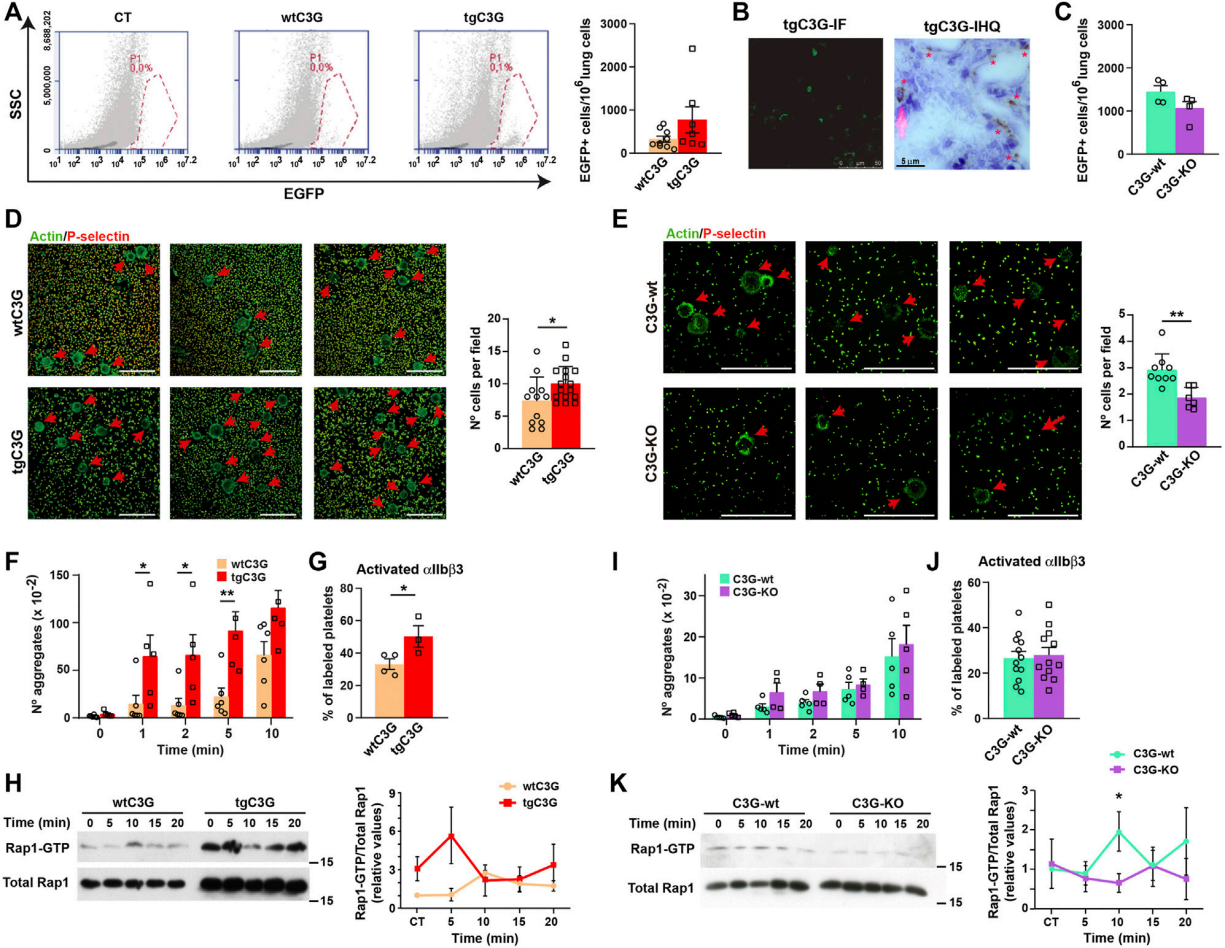
Platelet C3G favors an early onset of melanoma metastasis through regulation of melanoma cell adhesion and TCIPA. **(A)** Representative flow cytometry plots of lung homogenates from wtC3G and tgC3G animals injected with EGFP-expressing B16-F10 melanoma cells. CT: lung homogenates from untreated mice. Histogram represents the mean ± SEM of the number of EGFP^+^ cells in 1,000,000 lung cells. **(B)** Representative sections of lung tissue from tgC3G mice analyzed by immunofluorescence (IF, left) or immunohistochemistry (IHC) with anti-GFP antibodies (right). Presence of B16-F10 cells is indicated with asterisks. Bar: 5 μm. **(C)** Mean ± SEM of the number of EGFP^+^ cells in 1,000,000 lung cells from C3G-KO and C3G-wt mice. **(D,E)** Representative immunofluorescence images of B16-F10 cells adhered to poly-L-Lysine-coated plates, after co-culturing with tgC3G or wtC3G platelets **(D)** or C3G-KO and C3G-wt platelets **(E)**, stained with anti-P-selectin + anti-goat Cy3 antibodies (to visualize platelets) and phalloidin-488 to detect actin. Arrows indicate the presence of B16-F10 cells. Bar: 50 μm. Histograms represent the mean ± SD of the number of B16-F10 cells per field. **(F,I)** Mean ± SEM of the number of platelet aggregates from the indicated genotypes upon stimulation with B16-F10 cells for the indicated time periods. **(G,J)** Mean ± SEM of the percentage of platelets with activated integrin αIIbβ3. **(H,K)** Platelets from the indicated genotypes were stimulated with 1 × 10^3^ B16-F10 cells and Rap1-GTP levels determined by pull-down assay. Left: representative western blots. Right: line/scatter plots of Rap1 levels (n=3). Values (mean ± SEM) are relative to those in non-stimulated wild-type platelets and were normalized to total Rap1 levels. *p < 0.05, **p < 0.01.

Platelet C3G stimulates long-term lung metastasis of B16-F10 melanoma cells, in agreement with the higher angiogenesis observed in the implanted tumors (Martín-Granado et al., 2017). Based on that, we analyzed whether platelet C3G also participates in the initial adhesion and establishment of melanoma cells in the metastatic niche. Short-term metastasis analysis (Erpenbeck et al., 2010) revealed a more efficient arrest of GFP-labeled B16-F10 cells in the lungs when C3G is overexpressed in platelets (Figure 3A), indicating a role of C3G in this process. The implantation of tumor cells in the lungs was confirmed by immunofluorescence and immunohistochemistry (Figure 3B). This was supported by the lower number of GFP-expressing B16-F10 cells in lung homogenates from C3G-KO mice (Figure 3C).

The above results suggest that transgenic expression of C3G in platelets may potentiate the adhesive properties of melanoma cells. Indeed, B16-F10 cells showed a significant higher adhesion when co-cultured with tgC3G platelets (Figure 3D), while the opposite was found in cells co-cultured with C3G-KO platelets (Figure 3E).

These results support a role for platelet C3G in the early stages of metastasis, favoring tumor cell adhesion to the metastatic niche.

### Platelet C3G promotes TCIPA

We next studied the contribution of C3G to TCIPA. TgC3G platelets showed significantly greater aggregation than control platelets after incubation with B16-F10 cells (Figure 3F), which was accompanied by a higher activation of integrin αIIbβ3 (Figure 3G) and correlated with increased Rap1 activation (Figure 3H). We found no significant alterations in either TCIPA (Figure 3I) or integrin αIIbβ3 activation (Figure 3J) in C3G-KO platelets, although Rap1 activation was impaired (Figure 3K), as described in response to other stimuli (Gutiérrez-Herrero et al., 2020).

These results support the notion that C3G contributes to platelet-mediated tumor growth and metastasis, including cell adhesion to the metastatic niche and platelet-tumor cell communication, in agreement with previous findings (Martín-Granado et al., 2017).

### C3G ablation promotes an increase in the number of platelets after TPO stimulation

Another factor linking platelets to malignancy is TPO, whose levels are elevated in the serum of patients with various solid tumors, including lung adenocarcinoma (Werynska et al., 2003). TPO is the main cytokine that regulates megakaryopoiesis and we have previously described the participation of C3G in different steps of this process (Ortiz-Rivero et al., 2018). Strikingly, neither the overexpression nor the absence of C3G in platelets modified MK/platelet counts or platelet parameters under physiological conditions (Gutiérrez-Herrero et al., 2012; Ortiz-Rivero et al., 2018), Supplementary Figures S2A,B; Supplementary Table S4). However, platelet count increased in tgC3G mice after injection of TPO (Ortiz-Rivero et al., 2018), which suggests a contribution of C3G to pathological megakaryopoiesis.

**FIGURE 4:**
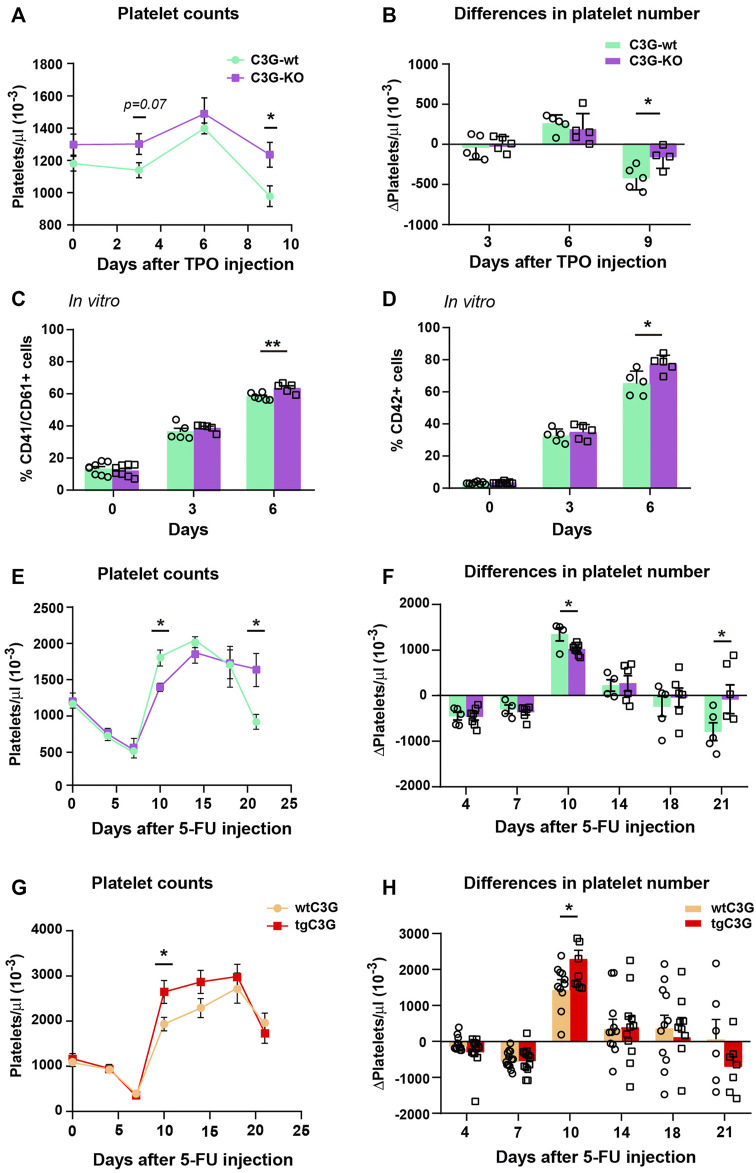
C3G regulates platelet levels after TPO injection or 5-FU-induced platelet rebound. **(A)**. Line/scatter plots of the mean ± SEM of platelet count in C3G-KO and C3G-wt mice at the indicated time points after TPO injection (n=9 C3G-KO and C3G-wt). **(B)** Differences in platelet number between two consecutive measurements in each genotype, as an indication of the increased or decreased rate. **(C,D)** Mean ± SD of the percentage of total (CD41^+^/CD61^+^) MKs **(C)** or mature (CD42^+^) MKs **(D)** in BM cultures of C3G-KO and control mice, at the indicated time points. **(E,G)** Line/scatter plots of the mean ± SEM of platelet count in C3G-KO and C3G- wt mice **(E)** or in tgC3G and wtC3G mice **(G)** at the indicated time points after 5-FU-injection (n=8 C3G-KO and C3G-wt; n=12 tgC3G, n=13 wtC3G). **(F,H)** Differences in platelet number between two consecutive measurements in each genotype, as an indication of the increased or decreased rate. *p < 0.05, **p < 0.01.

We used our C3G-KO mouse model to further investigate the putative role of C3G in megakaryopoiesis and thrombopoiesis. After intravenous injection of TPO, C3G-KO mice showed a greater increase in platelet levels than control animals (Figure 4A). In addition, after reaching the peak, C3G-KO mice failed to downregulate platelet levels (Figures 4A,B). No differences were found in the number of MKs of the treated mice or in their ploidy status (Supplementary Figure S2C). However, we observed a slightly, but significant, increase in the percentage of mature MKs in BM cultures of C3G-KO mice, after 6 days of stimulation with TPO plus a cocktail of cytokines (Figures 4C,D), which is consistent with the increased platelet counts detected *in vivo*.

### C3G-KO mice failed to recover homeostatic platelet levels after 5-FU-induced myelosuppression

Next, we studied C3G contribution to platelet rebound after 5-FU-induced myelosuppression. 5-FU induces platelet depletion around day 7 after injection, which is followed by a profound platelet rebound 10–15 days after treatment (Li and Slayton, 2013). Platelet rebound was slower in C3G-KO mice compared to C3G-wt siblings (Figures 4E,F). In addition, after peaking, platelet levels remained elevated in C3G-KO mice, indicating an impaired downregulation. Opposite results were obtained in tgC3G mice (Figures 4G,H). Differences were not due to changes in platelet half-life [(Ortiz-Rivero et al., 2018) and data not shown], nor to differences in the percentage of BM MKs or in their maturation (Supplementary Figures S2D,E).

Overall, these results suggest that C3G could be required to maintain homeostatic platelet levels.

### C3G regulates c-Mpl levels

**FIGURE 5:**
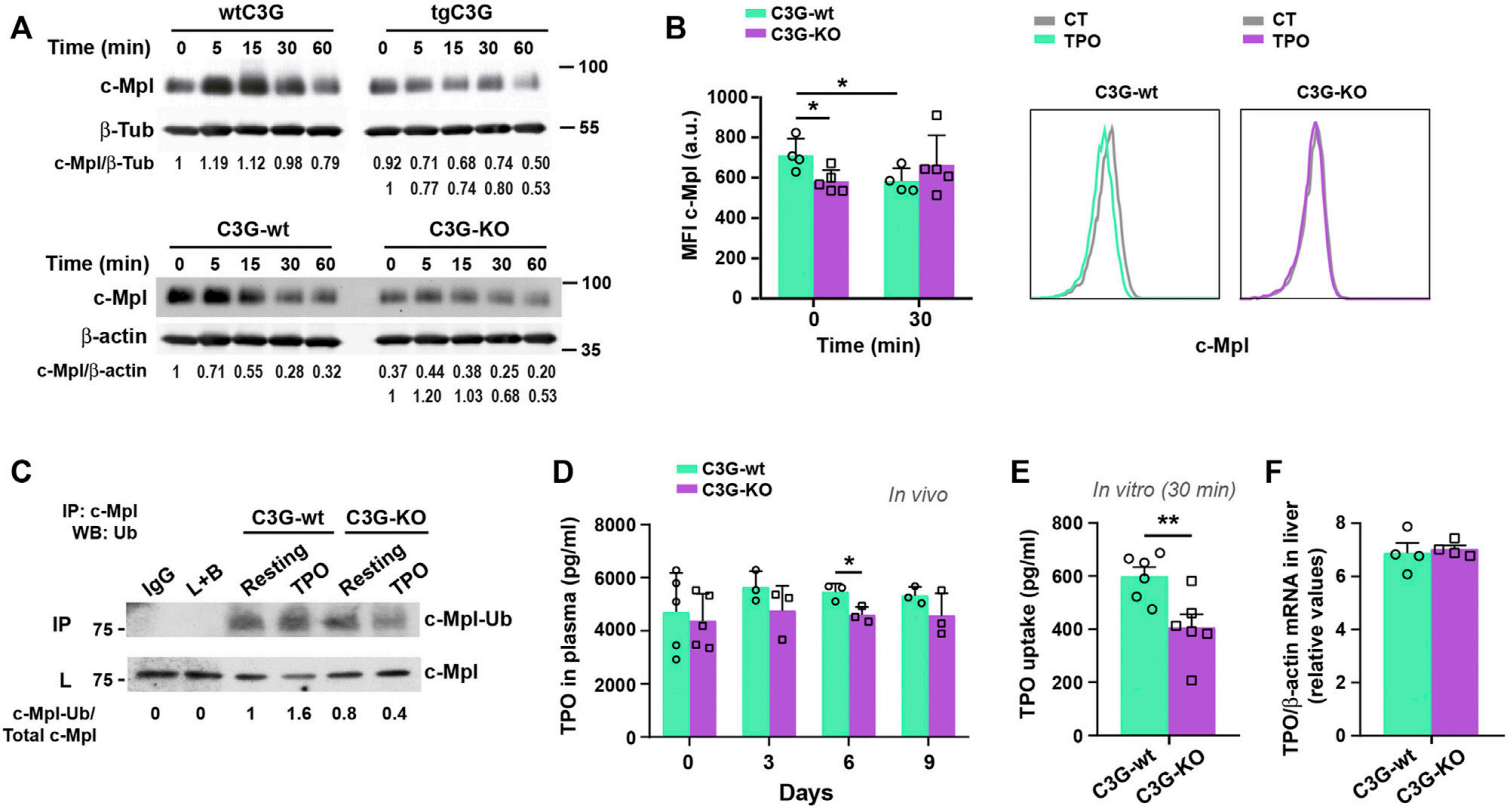
C3G regulates c-Mpl internalization and degradation. **(A)** Platelets from the indicated genotypes were stimulated with 25 ng/ml (tgC3G lineage) or 100 ng/ml (C3G-KO lineage) TPO for the indicated times and c-Mpl and β-tubulin (β-Tub) or β-actin detected by western blot. Values are relative to β-tubulin or β-actin expression and were normalized against untreated-wild-type platelets or against untreated platelets of each genotype (lower line). **(B)** Platelets of C3G-KO and control mice were treated 30 min with TPO and stained for c-Mpl surface expression with anti-c-Mpl (AMM2 clone) + anti-rat-Cy5 (Mean ± SD). Representative flow cytometry plots of c-Mpl internalization in each genotype are shown. CT: non-stimulated control. **(C)** Lysates from C3G-KO and C3G-wt platelets, resting or treated with TPO (100 ng/ml, 5 min were immunoprecipitated with anti-c-Mpl antibody and the levels of ubiquitinated-c-Mpl determined by western blot with anti-ubiquitin antibodies. IP: immunoprecipitation, WB: western blot. L: total cell lysates. IgG and lysate + agarose beads (L+B) were used as negative controls. Values are relative to those in non-stimulated C3G-KO platelets and were normalized to total c-Mpl. **(D)** C3G-KO and C3G-wt mice were injected with 0.5 μg TPO in 100 μl PBS. TPO was measured in plasma by ELISA at the indicated times (Mean ± SD). **(E)** C3G-KO and C3G-wt platelets were stimulated with TPO (2 ng/ml) for 30 min TPO levels in supernatant were measured by ELISA and TPO uptake was calculated as follows: TPO t0 pg/ml− TPO t30 pg/ml (Mean ± SEM). **(F)** TPO mRNA levels in livers from C3G-KO and C3G-wt mice were analyzed by RT-qPCR and normalized to β-actin (Mean ± SEM). *p < 0.*0*5, **p < 0.01. a.u, arbitrary units.

TPO promotes MK differentiation and platelet production *via* the TPO-c-Mpl signaling pathway (Kaushansky and Drachman, 2002). Since C3G appears to play a role in platelet maintenance, we investigated its involvement in the regulation of c-Mpl levels. C3G-KO platelets showed reduced levels of c-Mpl protein, while a small increase was found in tgC3G platelets, compared to wild-type ones (Supplementary Figure S3A). No differences were found in MKs (Supplementary Figure S3A). Moreover, while wild-type platelets exhibited a consistent time-dependent decrease in c-Mpl levels in response to TPO, tgC3G platelets showed a faster c-Mpl degradation than their controls. In contrast, C3G-KO platelets presented a delay in c-Mpl degradation (Figure 5A). This indicates that changes in the levels and/or functionality of C3G disrupt c-Mpl normal turnover. Furthermore, we found lower levels of c-Mpl on the surface of resting C3G-KO platelets and an impaired c-Mpl internalization in response to TPO (Figure 5B), a phenotype resembling that of c-Cbl-KO platelets (Marklin et al., 2020). The analysis of c-Mpl ubiquitination supported this. C3G-KO platelets showed significantly lower levels of ubiquitinated c-Mpl than control platelets, both at resting and in response to 5 min TPO (Figure 5C and Supplementary Figures S3B,C). Moreover, total ubiquitinated protein levels dropped drastically in resting, thrombin- (used as a positive control of ubiquitination) and TPO-stimulated C3G-KO platelets (Supplementary Figures S3B,C). This is supported by results in tgC3G platelets showing the opposite phenotype (Supplementary Figures S3D,E).

In addition, TPO injection resulted in a modest decrease in plasma TPO levels in C3G-KO mice (Figure 5D), which was in line with the enhanced platelet count observed in the same experimental settings (Figure 4A), according to the previously described inverse correlation between platelet and TPO levels (de Graaf and Metcalf, 2011). However, TPO uptake was significantly reduced in C3G-deficient platelets after 30 min incubation (Figure 5E), which correlates with the observed lower c-Mpl levels and its delayed internalization and degradation (Figures 5A,B). TPO mRNA expression in the liver was comparable in C3G-KO and C3G-wt mice (Figure 5F).

All this suggests that in C3G-KO platelets, the negative feedback regulation of c-Mpl signaling might be altered.

### C3G colocalizes with Cbl and facilitates its phosphorylation by SFKs

**FIGURE 6:**
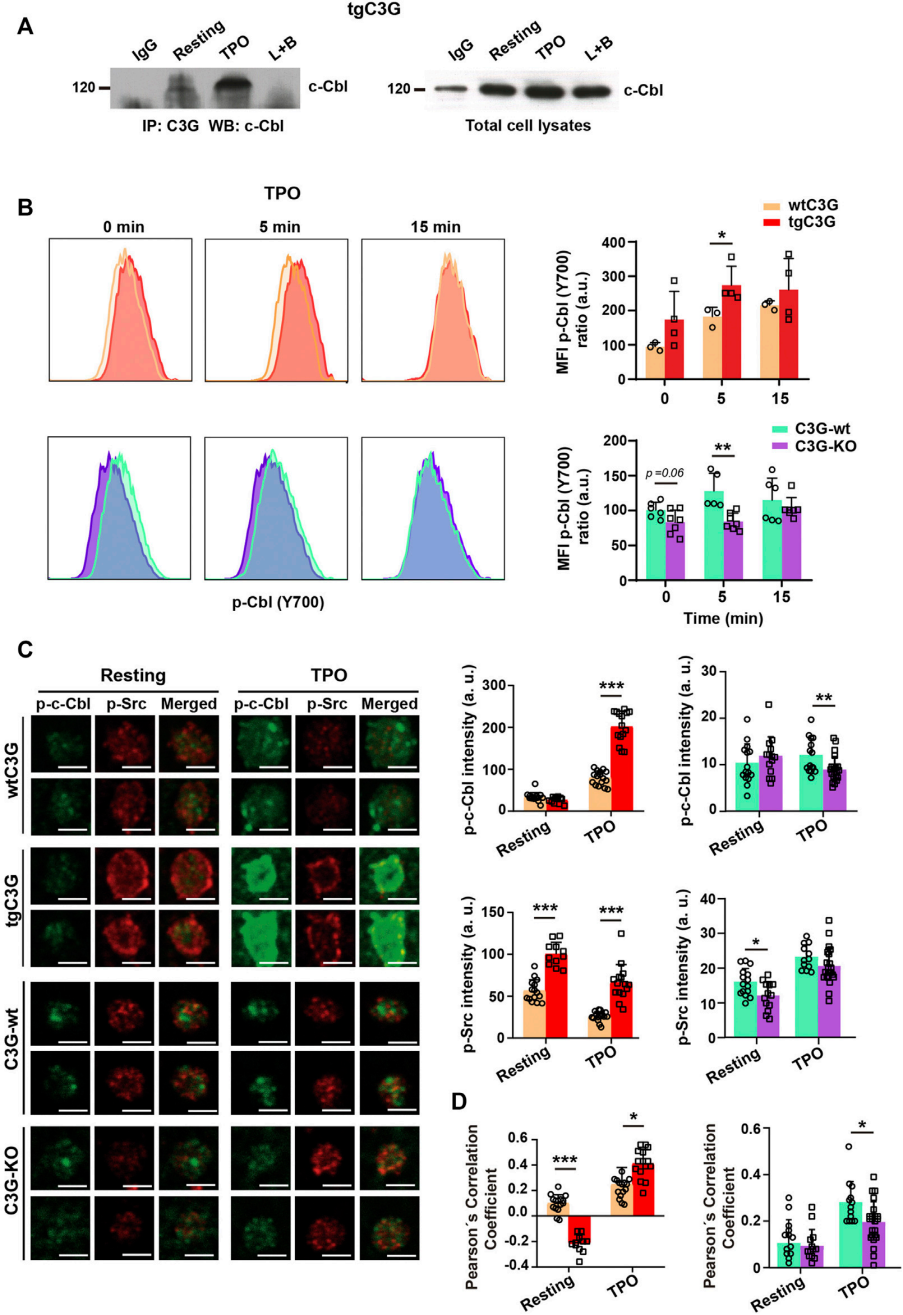
C3G interacts with c-Cbl and promotes c-Cbl phosphorylation by Src. **(A)** Lysates from tgC3G platelets, untreated or treated with TPO (100 ng/ml, 5 min were immunoprecipitated with anti-C3G antibodies and the levels of c-Cbl determined by western blot. IP: immunoprecipitation, WB: western blot. IgG and lysate + agarose beads (L+B) were used as negative controls. **(B)** tgC3G and C3G-KO platelets, and their controls were stimulated with TPO for the indicated time points. Intracellular phospho-c-Cbl levels were determined in fixed cells by flow cytometry, as described (Marklin et al., 2020). Left: representative flow cytometry plots. Right: Histograms represent the mean ± SD of the MFI value, calculated relative to the mean value of untreated wild-type controls. **(C)** Representative immunofluorescence images of platelets from the indicated genotypes, treated with TPO (100 ng/ml, 5 min and labeled with anti-phospho-c-Cbl + Alexa Fluor^TM^-647 (green) and anti-phospho-Src + Alexa Fluor^TM^-568 (red). All images were taken at the same exposure time. Bar: 2.5 μm. Histograms represent the mean ± SD of the fluorescence intensities of phospho-c-Cbl and phospho-Src. **(D)** Pearson’s Correlation Coefficients (mean ± SD) of phospho-c-Cbl and phospho-Src under the indicated experimental conditions. *p < 0.05, **p < 0.01, ***p < 0.001. a.u, arbitray units.

In platelets, c-Cbl is responsible for c-Mpl ubiquitination and degradation (Saur et al., 2010; Marklin et al., 2020). Therefore, we studied whether C3G participates in this c-Cbl function. As in other cell types (Reedquist et al., 1996; Maia et al., 2013), C3G and c-Cbl interact in platelets in response to agonists, mainly TPO (Figure 6A; Supplementary Figure S3F). Moreover, c-Cbl phosphorylation (analyzed by confocal immunofluorescence microscopy and flow cytometry, much more sensitive than western blotting and allowing a substantial reduction in the number of animals) was significantly increased in tgC3G platelets, whereas C3G ablation resulted in a decrease in phospho-c-Cbl levels, especially under thrombin or TPO stimulation (Figure 6B; Supplementary Figure S4A). Furthermore, we found higher p-Src levels (Figure 6C), and enhanced colocalization between p-Src and p-c-Cbl in TPO-stimulated tgC3G platelets, while the opposite effect was detected in C3G-KO platelets (Figures 6C,D), suggesting the participation of the three proteins in a complex. Supporting these findings, interaction between c-Cbl and Src has been previously reported (Song et al., 2010). Pretreatment with PP2, an inhibitor of Src family kinases (SFKs), further decreased c-Cbl phosphorylation and its colocalization with p-Src in C3G-KO platelets (Supplementary Figures S4B,C). PP2 also abolished the increase in p-c-Cbl levels observed in tgC3G platelets (Supplementary Figure S4D). These data indicate that C3G favors SFK-mediated phosphorylation of c-Cbl. Moreover, PP2 also inhibited TPO-induced c-Mpl ubiquitination, which was more evident in C3G-KO platelets, further confirming the additive effect of C3G and SFKs on c-Cbl activation (Supplementary Figures S3B–E).

All this points to the existence of a C3G-Src-c-Cbl pathway that leads to c-Mpl ubiquitination and degradation.

**FIGURE 7:**
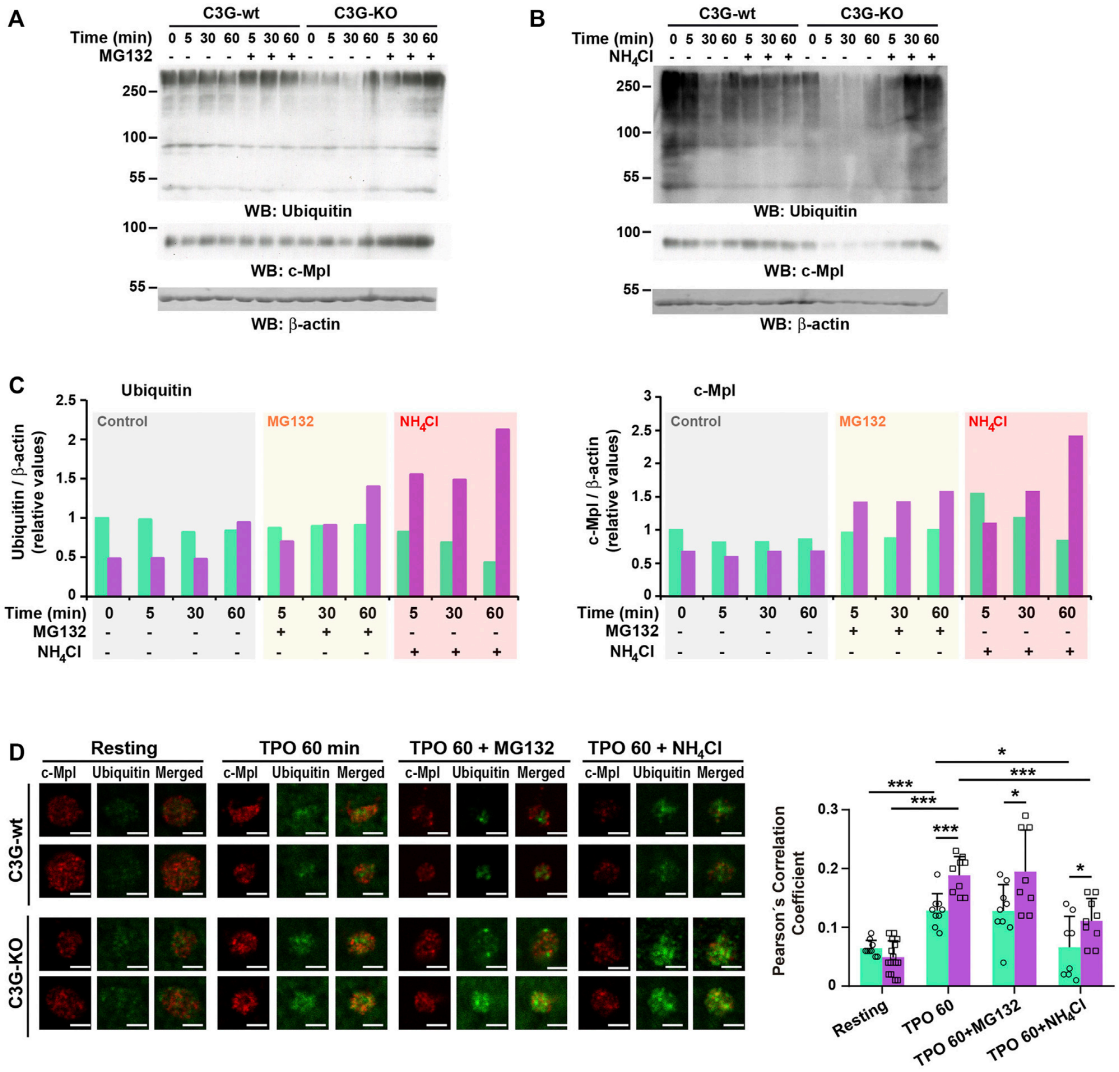
C3G plays a role in proteasome- and lysosome-mediated degradation of c-Mpl. Platelets from C3G-KO and C3G-wt mice were pre-treated 1 h with 30 μM MG132 **(A)** or 20 mM NH_4_Cl **(B)** prior incubation with 100 ng/ml TPO for the indicated times. The levels of ubiquitinated proteins and c-Mpl were measured by western blot using β-actin as loading control. **(C)** Histograms represent the quantification of ubiquitinated proteins (left panel) and c-Mpl levels (right panel) relative to β-actin and normalized against the t0 value of C3G-wt. **(D)** C3G-KO and C3G-wt platelets were treated with 100 ng/ml TPO for 60 min, after 1 h pre-treatment with 30 μM MG132 or 2 mM NH_4_Cl and labeled with anti-c-Mpl + Fluor^TM^-568 (red) or anti-Ubiquitin + Alexa Fluor^TM^-647 (green). Representative immunofluorescence images of platelets of each genotype under each treatment condition, taken at the same exposure time. Bar: 2.5 μm. The graph shows the Pearson’s Correlation Coefficients (mean ± SD) of c-Mpl and Ubiquitin under the indicated experimental conditions. *p < 0.05, ***p < 0.001.

In platelets, TPO-c-Cbl-mediated c-Mpl ubiquitination results in its destruction *via* the proteasome and lysosome systems (Saur et al., 2010; Marklin et al., 2020). We investigated whether C3G could affect these processes by analyzing total ubiquitination and c-Mpl levels in the presence of the proteasome inhibitor MG132. As expected, MG132 prevented, at least in part, TPO-induced decline in c-Mpl levels in wild-type platelets, which correlates with an accumulation of ubiquitinated proteins (Figures 7A,C). This effect was more pronounced in C3G-KO platelets, suggesting that the lysosome degradation mechanism may be hampered. Inhibition of lysosome with NH_4_Cl also induced a higher accumulation of ubiquitinated proteins and c-Mpl in C3G-KO platelets (Figures 7B,C). The fact that in both experiments the accumulation of c-Mpl coincided with that of ubiquitination suggests that c-Mpl is one of the ubiquitinated proteins (Figures 7A–C). Immunofluorescence analysis revealed higher ubiquitinated-c-Mpl levels in C3G-KO platelets treated with MG132 or NH_4_Cl after 60 min of TPO treatment, which supports these findings (Figure 7D).

These data suggest that C3G plays a role in proteasome- and lysosome-mediated degradation of c-Mpl.

## Discussion

We previously demonstrated that transgenic C3G expression in platelets promotes tumor-induced angiogenesis (Martín-Granado et al., 2017). Surprisingly, we found that C3G ablation in platelets also promoted angiogenesis. In fact, both, transgenic expression or absence of C3G in platelets induce proangiogenic secretomes, leading to increased vascular density in the implanted tumors (Martín-Granado et al., 2017) and a faster vascular regeneration in the hindlimb ischemia model, although, apparently, through different mechanisms. C3G overexpression resulted in retention of VEGF, SDF-1 and TSP-1, with retention of TSP-1 being responsible for the proangiogenic nature of the secretome, as described by us (Martín-Granado et al., 2017) and others (Feng et al., 2011). On the other hand, C3G absence in platelets also blocked TSP-1 secretion, but this was accompanied by a higher release of VEGF and SDF-1. This suggests that C3G may inhibit the release of proangiogenic factors, whereas the proper release of antiangiogenic factors may be affected by changes in normal C3G levels. Platelet C3G has been implicated in the differential secretion of pro- and antiangiogenic factors, which is thought to be related to its interaction with VAMP-7 (Martín-Granado et al., 2017), a v-SNARE protein essential for proper α-granule exocytosis (Peters et al., 2012; Koseoglu et al., 2015). Our findings indicate that distinct regulatory mechanisms would be involved in the release of pro- and antiangiogenic factors, which is consistent with the storage of these factors in different populations of α-granules within platelets, both in mice (Martín-Granado et al., 2017) and humans (Italiano et al., 2008; Chatterjee et al., 2011). PKC proteins, which mediate C3G phosphorylation in platelets (Gutiérrez-Herrero et al., 2020), regulate α-granule release by phosphorylating proteins of the secretory machinery (Harper and Poole, 2010). Specifically, PKCδ inhibits GPVI-dependent degranulation and release of proangiogenic factors, while it activates PAR-dependent secretion, mainly composed of antiangiogenic factors (Harper and Poole, 2010; Chatterjee et al., 2011). Therefore, PKC-C3G pathway might inhibit the release of the proangiogenic α-granules, whereas a separate signaling pathway also involving C3G, which deserves further investigation, would regulate the release of antiangiogenic α-granules.

Notably, although SDF-1 was retained in thrombin-stimulated tgC3G platelets, it accumulates primarily on the platelet surface; therefore it could also contribute to a more local recruitment of endothelial progenitor cells at the ischemic site (Massberg et al., 2006). Moreover, the increased levels of SDF-1 in the ischemic muscle of these mice could also explain the greater recovery of tgC3G mice under hindlimb ischemia. This extra SDF-1 present in ischemic muscle could be produced by enhanced vascular injury-induced thrombi (Massberg et al., 2006), since transgenic C3G promotes larger aggregation and thrombus formation (Gutiérrez-Herrero et al., 2012; Gutiérrez-Herrero et al., 2020). In contrast, no differences in SDF-1 expression in ischemic muscle were found between C3G-KO and wild-type mice, likely reflecting normal C3G-KO platelet aggregation activity, as occurs in response to melanoma cells. Furthermore, it has been reported that P-selectin present on the platelet surface is involved in the interaction with BM progenitor cells during vascular repair, and that integrin αIIbβ3 activation induces the exposure and release of SDF-1 (Massberg et al., 2006). Both, P-selectin and activated integrin αIIbβ3, are more abundant on the surface of tgC3G platelets, compared to wild-type platelets (Gutiérrez-Herrero et al., 2012).

On the other hand, differences in surface levels of P-selectin and activated integrin αIIbβ3 (Gutiérrez-Herrero et al., 2012; Gutiérrez-Herrero et al., 2020), both playing a role in platelet-tumor cell interaction (Chen and Geng, 2006; Erpenbeck et al., 2010) and the latter also contributing to TCIPA (Bambace and Holmes, 2011), are likely to be the cause of the antagonistic metastatic potential of tgC3G and C3G-KO platelets. The differential adhesion of melanoma cells co-cultured with tgC3G or C3G-KO platelets is in line with this conclusion. These results also support the previously reported involvement of platelet C3G in long-term B16-F10 cell lung metastasis (Martín-Granado et al., 2017). The lack of effect of C3G ablation on platelet activation and aggregation in response to melanoma cells is probably due to a compensatory mechanism driven by CalDAG-GEFI.

On the other hand, we have previously described that transgenic C3G expression enhances the ability of MKs to form proplatelets (Ortiz-Rivero et al., 2018), but without increasing platelet production in PB. Similar to what we found in tgC3G platelets, deletion of C3G did not influence the physiological platelet count, a phenotype similar to that of single Rap1a and Rap1b platelet knockouts (Stefanini et al., 2018).

Mechanistically, our data support a negative role of C3G in the TPO-c-Mpl-mediated signaling that regulates platelet levels, based on: 1) TPO induced increased platelet count in C3G-KO mice; 2) C3G null mice were unable to return to normal platelet levels following 5-FU-induced platelet rebound, which is also dependent on TPO (Li and Slayton, 2013), whereas tgC3G mice reached platelet levels below normal; 3) C3G interacts with c-Cbl and mediates its phosphorylation by Src or other SFKs; 4) as a consequence, ubiquitination of c-Mpl was enhanced in tgC3G platelets and impaired in C3G-KO platelets; 5) C3G-KO platelets showed lower levels of c-Mpl on the surface and a deficient internalization, indicating altered c-Mpl trafficking and turnover. Yet, a similar phenotype was described for the PF4-c-Cbl-KO mouse model (Marklin et al., 2020).

Deficient TPO-c-Mpl internalization in TPO-stimulated C3G-KO platelets could lead to enhanced c-Mpl signaling in MKs and, consequently, improved megakaryopoiesis, which could explain the increased platelet count in C3G-KO mice upon TPO injection. However, megakaryopoiesis requires an intact TPO-Cbl-C3G-Rap1 pathway in MKs (Garcia et al., 2001; Stork and Dillon, 2005); therefore, we speculate that other Rap1 GEFs, such as CalDAG-GEF1, could take on the role of C3G in this context. Supporting this idea, we found a rise in CalDAG-GEF1 levels in C3G-KO MKs (data not shown).

Inhibition of proteasome or lysosome degradation systems induced a strong accumulation of ubiquitinated proteins in C3G-KO platelets, indicating that C3G might be required for proper proteasome and lysosome activity. According to this, unpublished results from the group have detected a significant downregulation of Rpn1 expression in C3G-KO platelets. Rpn1 is a key subunit of the 19S proteasome regulatory particle, essential for proteasome assembly and function (Liu et al., 2020). A similar disruption of proteasome/lysosome-mediated degradation of c-Mpl has been associated with a loss of c-Cbl activity (Plo et al., 2017). The involvement of C3G in platelet proteasome and lysosome function will be explored in future research.

## Conclusion

In conclusion, in this work we present evidence for the involvement of C3G in ischemia-induced platelet-dependent angiogenesis, as well as additional data corroborating prior findings on the role of platelet C3G in melanoma cell lung metastasis. In addition, our data indicate that platelet C3G could play an important role in pathological megakaryopoiesis. This is supported by the increased platelet counts observed in C3G-KO mice upon TPO injection or 5-FU-induced myelosuppression. The fact that neither C3G overexpression nor ablation modifies physiological platelet count is compatible with a dual role of C3G: it would induce c-Mpl degradation in platelets, regulating TPO levels, and would participate in c-Mpl signaling in MKs leading to platelet production. Therefore, similar to c-Mpl (Ng et al., 2014), a tight regulation of C3G expression in platelets and MKs could play a role in the prevention of megakaryocytosis, thrombocytosis and myeloproliferative disorders.

## Data Availability

The original contributions presented in the study are included in the article/Supplementary Material, further inquiries can be directed to the corresponding authors.
