# Supplementary material for: New functions of C3G in platelet biology: Contribution to ischemia-induced angiogenesis, tumor metastasis and TPO clearance
Source: Front Cell Dev Biol. 2022 Oct 31;10:1026287. doi: 10.3389/fcell.2022.1026287 (PMC9661425; doi:10.3389/fcell.2022.1026287)
Supplement: Supplementary file 1 [file DataSheet1.pdf]

Supplementary Material

New functions of C3G in platelet biology: contribution to ischemia-induced angiogenesis, tumor metastasis and TPO clearance.

Luis Hernández-Cano[1,2], Cristina Fernández-Infante[1,2], Óscar Herranz[1,2], Pablo Berrocal[1,2], Francisco S. Lozano[2,3], Manuel A. Sánchez-Martín[2,4,5], Almudena Porras[6,7]* and Carmen Guerrero[1,2,5]*

[1]Instituto de Biología Molecular y Celular del Cáncer (IMBCC), USAL-CSIC.
[2]Instituto de Investigación Biomédica de Salamanca (IBSAL), Salamanca, Spain.
[3]Departamento de Angiología y Cirugía Vascular, Hospital Universitario de Salamanca, Universidad de Salamanca, Salamanca, Spain
[4]Servicio de Transgénesis, Nucleus, Universidad of Salamanca, Salamanca, Spain
[5]Departamento de Medicina, Universidad de Salamanca, Salamanca, Spain
[6]Departamento de Bioquímica y Biología Molecular, Facultad de Farmacia, Universidad Complutense de Madrid, Madrid, Spain.
[7]Instituto de Investigación Sanitaria del Hospital Clínico San Carlos (IdISSC), Madrid, Spain.

*Correspondence: C. Guerrero, Centro de Investigación del Cáncer, Campus Unamuno s/n, Salamanca, Spain. Tel.: +34 923294801; e-mail: cguerrero@usal.es. Co-correspondence: A. Porras, Departamento de Bioquímica y Biología Molecular, Facultad de Farmacia, UCM, Ciudad Universitaria; IdISSC, Madrid, Spain. Tel.: +34 913941627; e-mail: maporras@ucm.es

- Supplementary Tables S1-S4
- Supplementary Figures S1-S4

Table S1. Genetically modified animals

Species (mouse)	Source	EMMA Mouse repository	Background strain
tgPF4-C3G	In house generated<br>PMID: 22659131	EM:06334	C57BL/6J
Rapgef1$^{flox/flox}$;PF4-Cre$^{+/-}$<br>Rapgef1$^{flox/flox}$;PF4-Cre$^{-/-}$	In house generated<br>PMID: 32296045		C57BL/6J

Table S2. PCR primers

Target	Forward	Reverse
β-actin	TAGACTTCGAGCAGGAGATGG	CAAGAAGGAAGGCTGGAAAG
VEGFA	GAGAGAGGCCGAAGTCCTTT	TTGGAACCGGCATCTTTATC
CD31	ACTTCTGAACTCCAACAGCGA	CCATGTTCTGGGGGTCGTAAT
SDF-1	AGCCAACGTCAAGCATCT	GCACACTTGTCTGTTGTTGTT
mTPO	TTCAGTGTCACAGCCAGAAC	GGGACCTGGAGGTTTGATTTAG

Table S3. Antibodies used for flow cytometry, confocal immunofluorescence microscopy, immunohistochemistry, immunoprecipitation and western blot.

Target antigen	Vendor or Source	Catalog #	Working concentration
**Flow cytometry**			
PE Anti-Mouse CXCR4<br>(#247506 clone)	R&D Systems	FAB21651P	1:50
APC Anti Mouse VEGFR<br>(#141522 clone)	R&D Systems	FAB4711A	1:50
FITC Anti-Mouse CD41<br>(MWReg30 clone)	eBiosciences	11-0411-82	1:50
PE Anti-Mouse CD61<br>(2C9.G3 clone)	eBiosciences	12-0611	1:50
FITC GPIalpha (CD42b)	Emfret Analytics	M040-1	1:50
Anti-mouse c-MPL/TPOR /AMM2)<br>RatIgM MoAb	Tecan	JP10401	1:50
**Confocal immunofluorescence microscopy**			
**Primary**			
VEGF	Abcam	ab1316	1:200
TSP-1	Thermo Scientific	MA5-13398	1:200
SDF-1	R&D Systems	MAB350-100	1:100
P-selectin (C-20)	Santa Cruz Biotechnology	sc-6941	1:100
C3G #1008	Guerrero et al., 1998		1:50
c-Cbl	Cell Signalling	2747	1:100
phospho-c-Cbl (E-10)	Santa Cruz Biotechnology	sc-377571	1:100
phospho-Src Y418	Abcam	ab4816	1:100
c-Mpl	Merck	06-944	1:100
Ubiquitin (P4D1)	Santa Cruz Biotechnology	sc-8017	1:100

**Secondary**			
Alexa Fluor™-568-conjugated Goat anti-rabbit	Invitrogen	A-11036	1:500
Alexa Fluor™-647-conjugated Goat anti-mouse	Invitrogen	A-21236	1:500
Cy™3-Affinipure donkey Anti-goat IgG	Jackson Immunoresearch	705-165-147	1:100
Cy™5 AffiniPure donkey Anti-rat IgG	Jackson Immunoresearch	111-175-144	1:100
**Markers**			
Phalloidin-iFluor 488	Abcam	ab176753	1:2000
**Immunohistochemistry**			
GFP (FL)	Santa Cruz Biotechnology	sc-8334	1:50
CD31	Abcam	ab28364	1:50
**Western blot**			
VEGF	Abcam	ab1316	1:500
TSP-1	Thermo Scientific	MA5-13398	1:1000
c-Cbl	Cell Signalling	2747	1:1000
Rap1	Santa Cruz Biotechnologies	sc-65	1:1000
c-Mpl	Merck	06-944	1:1000
Ubiquitin (P4D1)	Santa Cruz Biotechnology	sc-8017	1:500
β-actin	Merck	A5441	1:1000
β-tubulin	Merck	T5293	1:1000
**Immunoprecipitation**			
c-Mpl	Merck	06-944	1:50
C3G (G-4)	Santa Cruz Biotechnologies	sc-17840	1:50

Table S4. Deletion of C3G did not modify platelet counts and its parameters. Platelet number and parameters in C3G-KO mice and their C3G-wt siblings, male and female. The counts were made using an Advia 120 Hematology Analyzer (Bayer). Values are the mean of five, 10-week, mice of each genotype. There were no significant differences between genotypes or genders.

Parameter	Units	C3G-wt male	C3G-KO male	C3G-wt female	C3G-KO female
Platelet count	10^3 cells/μl	1259.2	1179.4	1092	1160.25
MPV (Mean platelet volume)	fL	6.4	6.38	6.56	6.375
PDW (Platelet distribution width)	%	51.08	53.68	53.58	49.225
PCT (plateletcrit)	%	0.81	0.752	0.718	0.74
MPC (Mean platelet component)	g/dl	21.74	21.94	21.78	22.125
PCDW (Platelet component distribution width)	g/dl	6.9	7.14	7.12	7.05
MPM (Mean platelet (dry) mass)	pg	1.24	1.234	1.266	1.2675
PMDW (Platelet mass distribution width)	pg	0.422	0.428	0.428	0.4225
Large PLT	10^3 cells/μl	5	5.6	5	3
RBC fragments	10^6 cells/μl	0.166	0.162	0.142	0.15
RBS ghosts	10^6 cells/μl	0.05	0.044	0.056	0.055
Clumps count		44.6	42.2	111.2	51.5

Figure S1

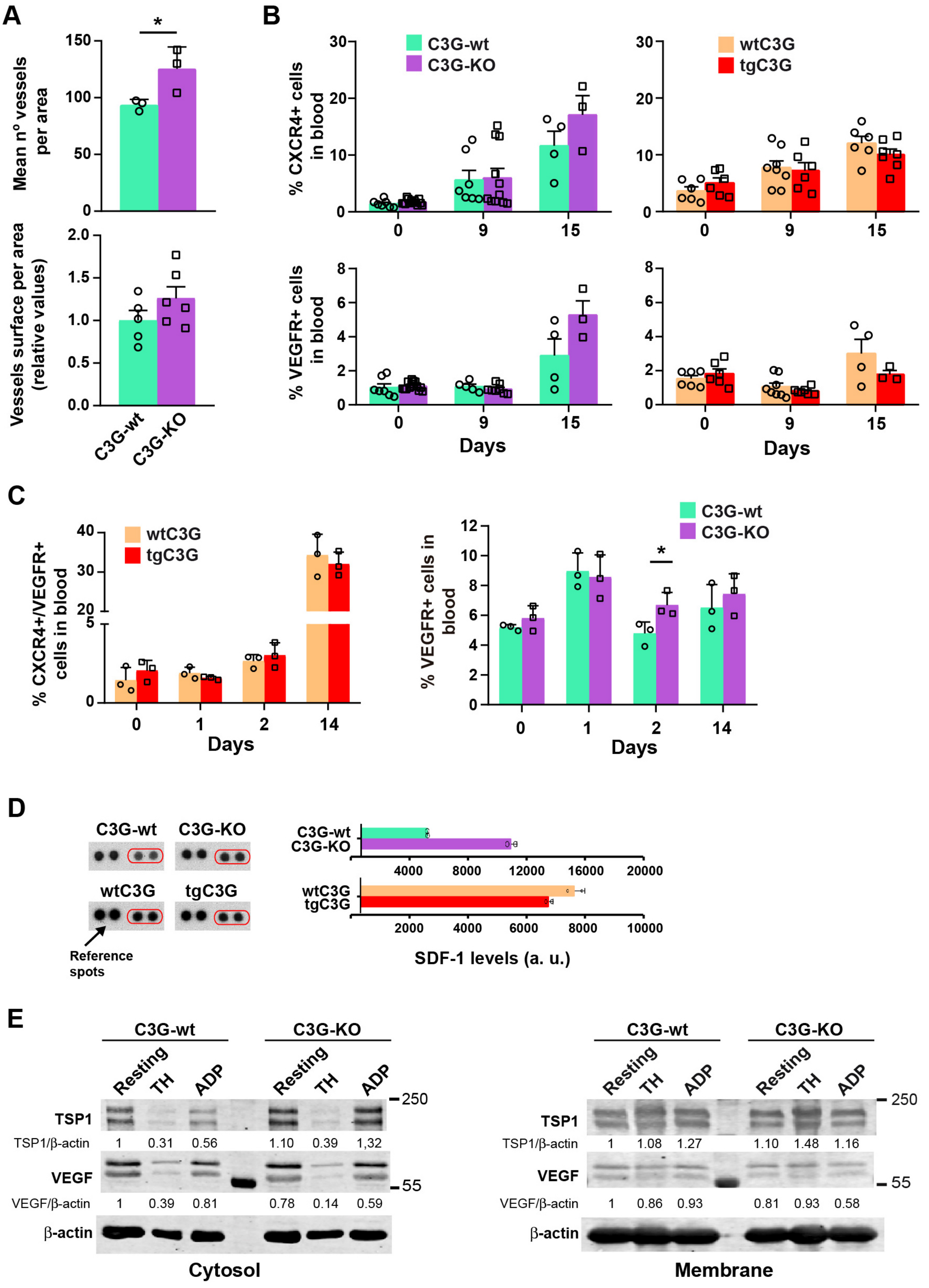

Figure S1. Platelet C3G regulates ischemia-induced angiogenesis. (**A**) 3LL cells were injected in C3G-KO mice and their controls and tumors removed after 15 days. Histograms represent the number of vessels per area (upper) and the vessels surface per area (lower) (mean ± SEM) in tumor sections. (**B**) Blood collected at the indicated days

after implantation of 3LL cells in tgC3G, C3G-KO mice and their controls was incubated with anti-CXCR4-PE and anti-VEGFR-APC to determine the percentage of hemangiocytes. Histograms represent the mean ± SEM of the percentage of CXCR4$^+$ (upper) or VEGFR$^+$ (lower) cells in peripheral blood from the indicated genotypes. (**C**) Histograms represent the mean ± SEM of the percentage of CXCR4/VEGFR-double positive cells in blood from tgC3G mice and their controls (left) and the percentage of VEGFR-positive cells in blood from C3G-KO mice and their controls (right), collected at the indicated times post-ischemia. (**D**) Histogram represents the quantification of SDF-1 levels in thrombin-induced secretome from tgC3G, C3G-KO platelets and their controls, using a Mouse Angiogenesis Array Kit (n=2, each per duplicated). Representative images of the arrays are depicted in the left panels. SDF-1 spots are marked with red boxes. a. u, arbitrary units. Reference spots, which are not suitable for quantification, are indicated. (**E**) Western blot analysis of TSP-1 and VEGF levels in cytosolic (left) or membrane (right) fractions from resting, thrombin (TH)- or ADP- stimulated C3G-KO or C3G-wt platelets. β-actin was used as loading control. Values were normalized to those of resting C3G-wt platelets. *$p<0.05$.

Figure S2

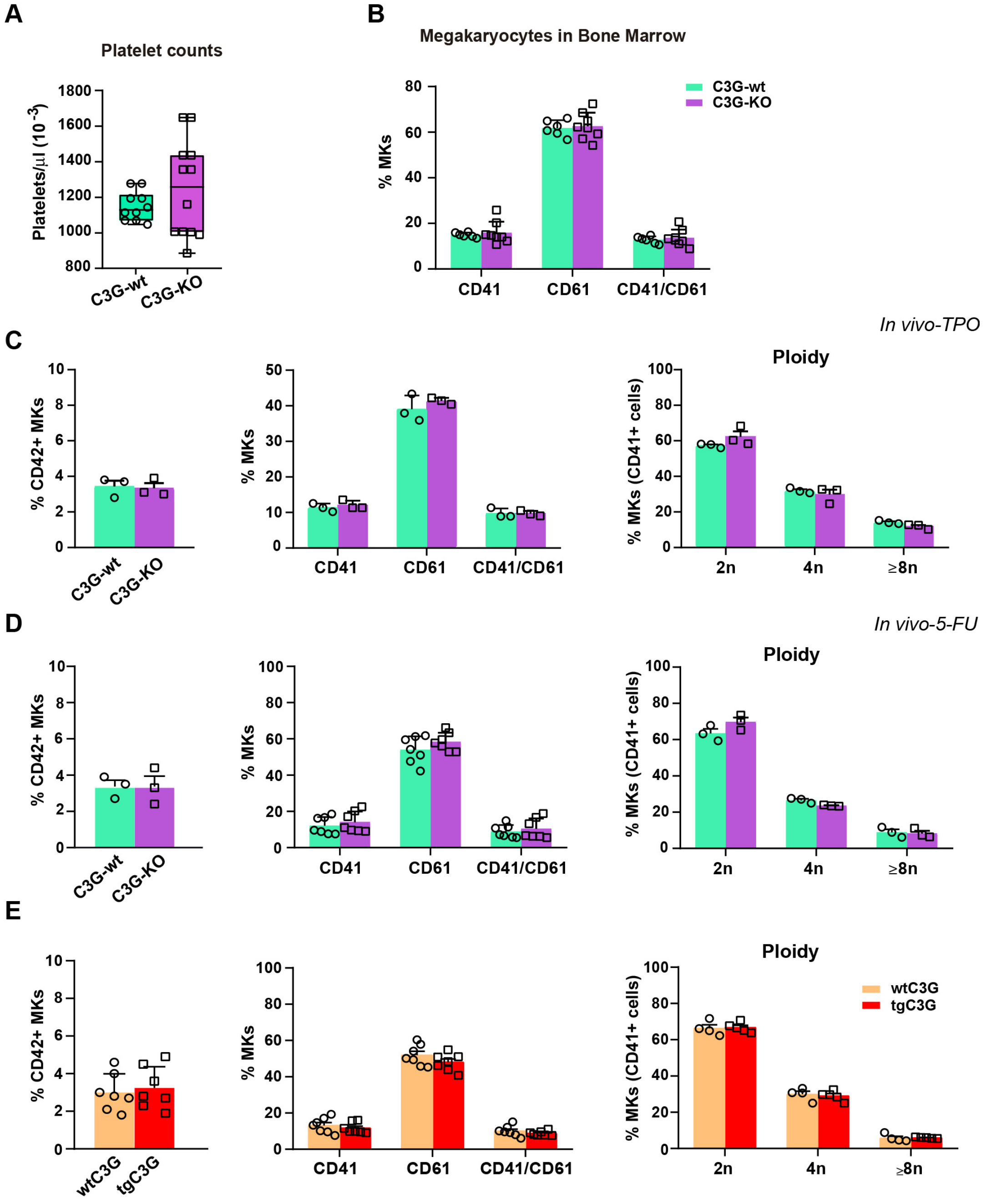

Figure S2. C3G ablation or overexpression does not modify physiological MK or platelet production, nor does MK production after TPO injection or 5-FU-induced BM depletion. (**A**) Box plots showing the median of the number of platelets in C3G-KO mice and their control siblings. (**B**) Expression of CD41 and CD61 markers in BM cells from the indicated genotypes was determined by flow cytometry using CD41-FITC and CD61-PE. Histograms represent the mean ± SD of the percentage of CD41+, CD61+ or double-positive cells (megakaryocytes). (**C**) Expression of CD42 (left), CD41 and CD61 markers (middle) and ploidy status (right) was analyzed by flow cytometry in BM from C3G-KO and control mice, 14 days after injection of TPO. (**D, E**) Expression of CD42 (left), CD41 and CD61 markers (middle) and ploidy status (right) was analyzed by flow cytometry in BM from C3G-KO and C3G-wt mice (**D**) or tgC3G and wtC3G mice (**E**), 21 after 5-FU injection.

Figure S3

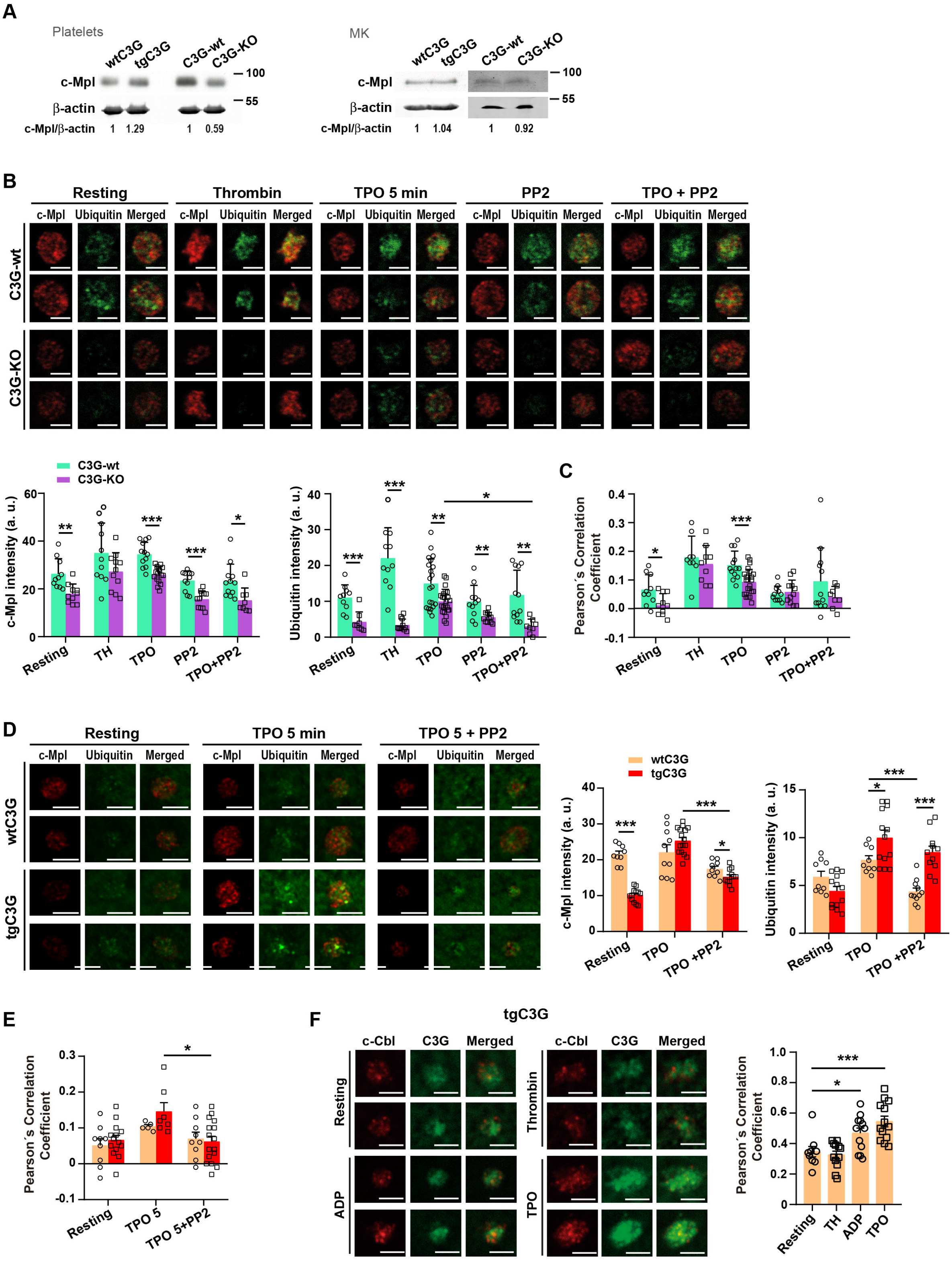

Figure S3. **C3G regulates c-Mpl levels and its ubiquitination and interacts with c-Cbl.** (**A**) Western blot analysis of c-Mpl protein levels in platelets (left) or MKs (right) from the indicated genotypes. Values are relative to β-actin expression and were normalized

against those of each wild-type. (**B**) C3G-KO and C3G-wt platelets were treated with thrombin (TH, 0.5 U/ml, 1 min) or TPO (100 ng/ml, 5 min) in the presence or absence or the SFK inhibitor PP2 (10 μM) and labeled with anti-c-Mpl + Alexa Fluor™-568 (red) or anti-Ubiquitin + Alexa Fluor™-647 (green). Upper panels: representative immunofluorescence images of platelets taken at the same exposure time. Bar: 2.5 μm. Histograms represent the mean ± SD of the fluorescence intensities of c-Mpl (left) or ubiquitin (right). (**C**) The graph shows the Pearson's Correlation Coefficients (mean ± SD) of c-Mpl and ubiquitin under the indicated experimental conditions. (**D**) tgC3G and wtC3G platelets were treated with TPO (100 ng/ml, 5 min) in the presence or absence of the SFK inhibitor PP2 (10 μM) and labeled with anti-c-Mpl + Alexa Fluor™-568 (red) or anti-Ubiquitin + Alexa Fluor™-647 (green). Left panels: representative immunofluorescence images of platelets of each genotype under each treatment condition, taken at the same exposure time. Bar: 2.5 μm. Histograms represent the mean ± SD of the fluorescence intensities of c-Mpl (left) or ubiquitin (right). (**E**) The graph shows the Pearson's Correlation Coefficients (mean ± SD) of c-Mpl and ubiquitin under the indicated experimental conditions. (**F**) TPO induces C3G and c-Cbl colocalization. Representative immunofluorescence images of tgC3G platelets treated with TH (0.5 U/ml, 1 min), ADP (25 μM, 5 min) or TPO (100 ng/ml, 5 min) and labeled with anti-c-Cbl + Alexa Fluor™-568 (red) and anti-C3G + Alexa Fluor™-647 (green). Histograms show the Pearson's Correlation Coefficients (mean ± SD) of C3G and c-Cbl under the indicated experimental conditions. *$p<0.05$, **$p<0.01$, ***$p<0.001$. a.u, arbitrary units.

Figure S4

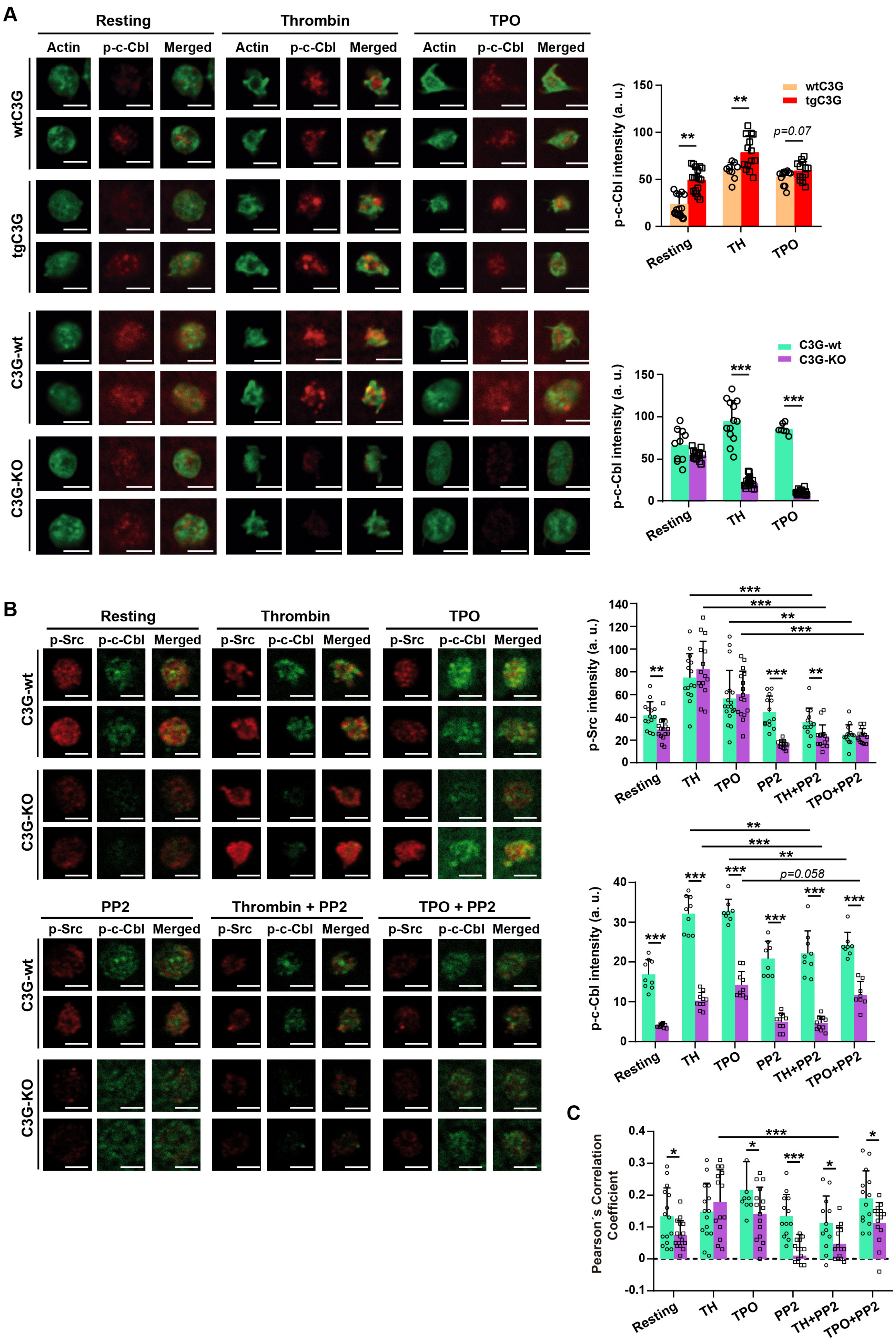
A
Resting
Thrombin
TPO
Actin
p-c-Cbl
Merged
wtC3G
tgC3G
C3G-wt
C3G-KO
p-c-Cbl intensity (a. u.)
wtC3G
tgC3G
p=0.07
Resting
TH
TPO
C3G-wt
C3G-KO
B
p-Src
PP2
Thrombin + PP2
TPO + PP2
p-Src intensity (a. u.)
p=0.058
TH+PP2
TPO+PP2
C
Pearson´s Correlation Coefficient

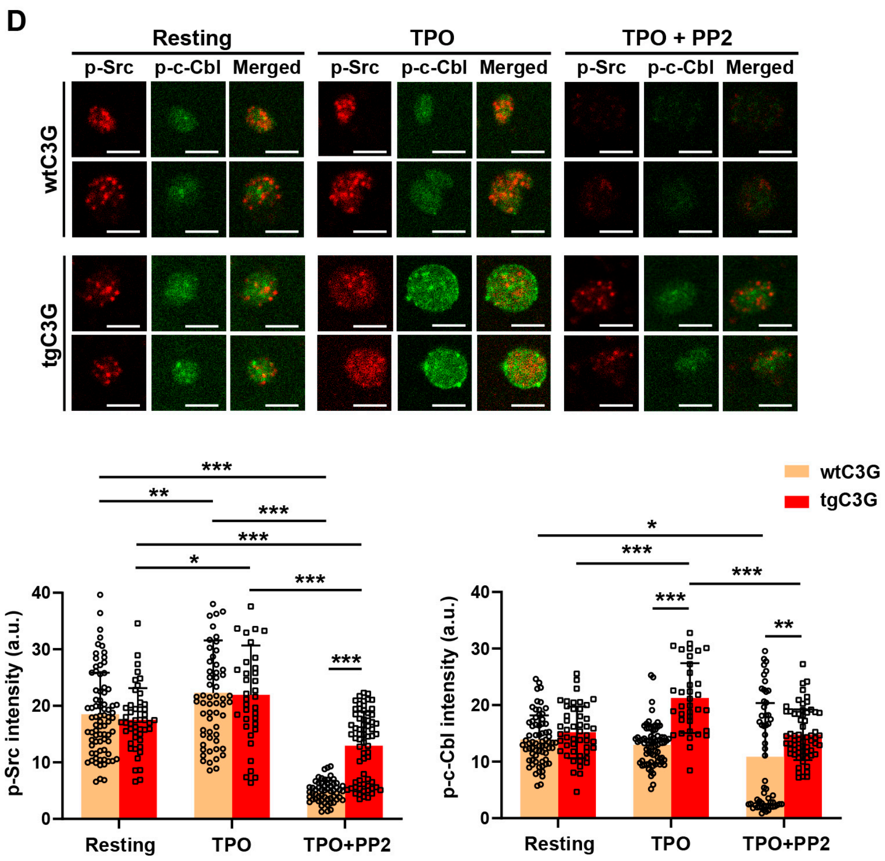

Figure S4. C3G promotes c-Cbl phosphorylation by Src. (**A**) Representative immunofluorescence images of tgC3G, C3G-KO and control platelets treated with thrombin (TH, 0.5 U/ml, 1 min) or TPO (100 ng/ml, 5 min) and labeled with anti-phospho-c-Cbl + Alexa Fluor^{TM}-647 (red) and Phalloidin (green). All images were taken at the same exposure time. Bar: 2.5 µm. Histograms represent the mean ± SD of the fluorescence intensities of phospho-c-Cbl (p-c-Cbl). (**B**) Representative immunofluorescence images of C3G-KO platelets and their controls treated with TH (0.5 U/ml, 1 min) or TPO (100 ng/ml, 5 min), in the presence or absence of PP2, and labeled with anti-phospho-c-Cbl + Alexa Fluor^{TM}-647 (green) and anti-phospho-Src + Alexa Fluor^{TM}-568 (red). All images were taken at the same exposure time. Bar: 2.5 µm. Histograms represent the mean ± SD of the fluorescence intensities of phospho-Src (p-Src) (upper) and phospho-c-Cbl (lower) under the indicated treatments. (**C**) Graph showing the Pearson's Correlation Coefficients (mean ± SD) of phospho-c-Cbl and phospho-Src under the indicated experimental conditions. (**D**) Representative immunofluorescence images of tgC3G platelets and their controls treated with TPO (100 ng/ml, 5 min), in the presence or absence of PP2, and labeled with anti-phospho-c-Cbl + Alexa Fluor^{TM}-647 (green) and anti-phospho-Src + Alexa Fluor^{TM}-568 (red). All images were taken at the same exposure time. Bar: 2.5 µm. Histograms represent the mean ± SEM of the fluorescence intensities of phospho-Src (p-Src) (left) and phospho-c-Cbl (right) under the indicated treatments. $^{*}p<0.05$, $^{**}p<0.01$, $^{***}p<0.001$. a.u, arbitrary units.
